# Physical and Structural Properties of Chitosan–Squid Gelatin Hydrogels

**DOI:** 10.3390/gels11020109

**Published:** 2025-02-03

**Authors:** Uriel Ramírez-Campas, Santiago P. Aubourg, Wilfrido Torres-Arreola, Maribel Plascencia-Jatomea, Josafat Marina Ezquerra-Brauer

**Affiliations:** 1 Department of Food Research and Graduate Studies, University of Sonora, Blvd. Luis Encinas y Rosales s/n, Col Centro, Hermosillo 83000, Sonora, Mexico; uriel_campas1990@hotmail.com (U.R.-C.); wilfrido.torres@unison.mx (W.T.-A.); maribel.plasencia@unison.mx (M.P.-J.); 2 Department of Food Technology, Marine Research Institute (CSIC), c/E. Cabello, 6, 36208 Vigo, Spain

**Keywords:** chitosan hydrogel, gelatin, squid by-products, antioxidant activity, physical properties, structural properties

## Abstract

The development of functional hydrogels is currently receiving great attention. In this study, a squid by-product, gelatin (SG)–acetic acid solution, was added to a commercial chitosan (CH)–acetic acid solution to develop an antioxidant hydrogel. The CH–SG mass ratios evaluated were 1:0, 2:1, and 1:2. Glutaraldehyde was used as cross linker. The effects of the SG addition to the hydrogel on different properties (physical in general, stability in aqueous media at pH 7.2, swelling, textural profile, and antioxidant) were evaluated. The interaction of CH and SG was established by scanning electron microscope microscopy (SEM), Fourier-transform infrared spectroscopy (FTIR), and nuclear magnetic resonance spectroscopy (NMR). As a result, the addition of SG decreased the resistance to flow, hardness, chewiness, and stability, but increased the springiness, resilience, and antioxidant properties of CH hydrogels. The SEM analysis revealed that the CH-GS hydrogel showed a relatively more porous structure. FTIR and NMR analyses suggested a good compatibility of the components due mainly to an increased hydrogen bond formation. The present results suggest that CH could establish a valuable interaction with SG, so that a new hydrogel with enhanced textural and antioxidant properties would be produced, which would enable its potential application in biomedical and food industries.

## 1. Introduction

Research focused on the development of functional natural materials, such as hydrogels, has received considerable attention over the past six decades [1]. In general, hydrogels have been defined as crosslinked polymer chains with a three-dimensional structure that exhibit the ability to absorb and retain a significant amount of water, attributable to the presence of hydrophilic groups, such as -COOH, -CONH_2_, -NH_2_, -OH, and -SO_3_H, among others [2]. Natural hydrogels can be constructed by connecting polysaccharides, like chitosan, and proteins, such as gelatin, by using crosslinkers [3]. Additionally, hydrogel versatility allows for their application for biomedical, food [2], and food packaging [4] purposes.

Among the most studied natural polymers in hydrogel designs, chitosan and gelatin can be mentioned. Chitosan is an amino polysaccharide chitin derivative compound, with a wide range of valuable applications [5]. Thus, chitosan has been studied extensively for its interesting physicochemical and biological properties, high biodegradability, and biocompatibility with other molecules [5]. One of its main applications is the production of biofilms and hydrogels [6]. However, when used alone, the obtained material presents a low surface area, negligible porosity, weak mechanical strength, and water insolubility. Notably, chitosan crosslinking with other biomaterials can lead to important modifications that can avoid such inconveniences [7].

Gelatin, derived from collagen through limited hydrolysis/heat denaturation, has a high water solubility and offers excellent film-forming properties and complexes with other molecules [8]. Chemically, gelatin is composed of about 18 different amino acids, with glycine, proline, and hydroxyproline being the most relevant [8]. Moreover, gelatin is a fat- and cholesterol-free molecule, shows a high protein content, and possesses protective colloid properties [8].

Moreover, the huge amount of underutilized seafood by-products is a critical problem worldwide in social, economic, and environmental terms. Therefore, many researchers have focused on the valorization of by-products resulting from commercial seafood processing [9]. Among marine species, Dosidicus gigas, known as jumbo squid, giant squid, or jumbo flying squid, has a high value due to its versatility in human nutrition and its low cost [10]. According to the Food and Agriculture Organization of the United Nations [11], the total marine capture of jumbo squid was approximately 1,076,428 tons in 2022, and more than 50% of the total organism weight was discarded, as fins, heads, tentacles, skin, and viscera were usually managed as waste [12].

Squid by-product gelatin is characterized as containing similar concentrations of proline and hydroxyproline as bovine collagen, presenting a high thermal transition temperature [13] and providing the possibility of reducing oxidative stress [14]. Moreover, it is known that the electrostatic attraction between the chitosan -NH_2_ group and the gelatin -COO groups may facilitate the crosslinking process [3]. Although some studies have focused on the biofilm properties of commercial chitosan blended with squid by-product collagen [15], there is no available literature regarding the properties of hydrogels composed of chitosan blended with gelatin extracted from squid by-products.

Consequently, this work is focused on the evaluation of the potential application of squid by-product gelatin as an additive during the preparation of chitosan–glutaraldehyde hydrogels. The objective of this study was to improve the antioxidant properties of chitosan hydrogel (H). The effects of two different concentrations of squid by-product gelatin on the physical, texture profile, and antioxidant properties of the chitosan–glutaraldehyde hydrogel were examined. Moreover, compatibility among the components was evaluated by Fourier-transform infrared spectroscopy and nuclear magnetic resonance spectrum analyses.

## 2. Results and Discussion

### 2.1. Steady Shear Measurements

**Figure 1:**
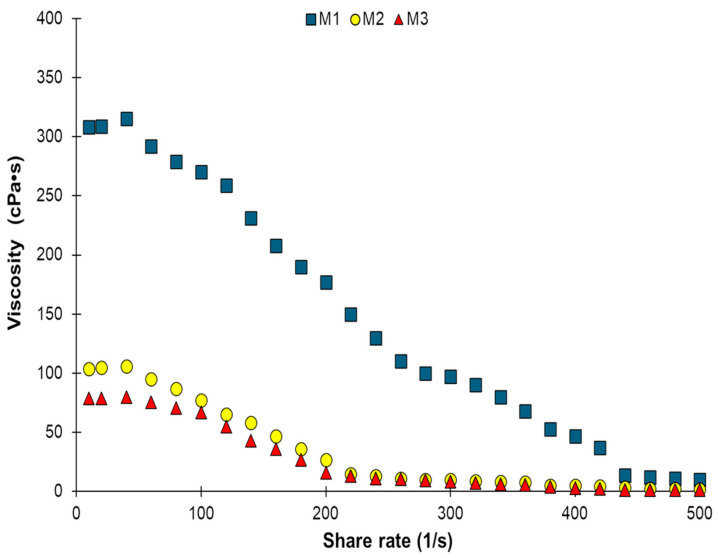
Viscosity (μ) of hydrogel-forming solutions: chitosan–squid gelatin mass ratios: 1:0 (M1), 2:1 (M2), and 1:2 (M3).

Viscosity is associated with biofilm particle stability and is important in establishing its potential applications. Most materials of technical and practical importance, such as chitosan and protein solutions, behave as typical non-Newtonian fluids because they do not exhibit a proportional relationship between the shear stress and the shear rate [16]. The apparent viscosity dependence on the shear rate of the different chitosan–squid by-product gelatin solutions is shown in Figure 1. The apparent viscosity of the three solutions decreased when gradually increasing the shear rate, and they exhibited a non-Newtonian shear-thinning behavior. With the increase in the shear rate, the dispersed molecules are orientated, causing a decrease in internal frictions due to a smaller effective interaction between molecules [16]. The initial viscosity of the M1 solution was higher than those of the M2 and M3 solutions (Table 1), but the M1 solution viscosity showed a more pronounced decrease (Figure 1). Thus, the initial viscosity values were 308.9 cPa·s, 103.9 cPa·s, and 78.6 cPa·s for the M1, M2, and M3 conditions, respectively. This behavior could be explained by the collagen molecules’ entanglement degree: when the chitosan decreases the freedom, the movement of the individual chain increases as a result of the decrease in molecule entanglements [17]. It has been demonstrated that the viscosity of chitosan solutions increases with the addition of gelatin due to the interaction formed between the two polymers [18]. The high levels of hydroxyproline and glycine and the amino and carboxyl groups present in the gelatin may decrease the repulsion between the charged chitosan chains of the molecule [19]. Moreover, the viscosity values of the three solutions were lower than 700 mPa·s, suggesting that the solutions are suitable for food-coating applications [18].

### 2.2. Stability

Stability in an aqueous medium is an important property related to the application of the prepared hydrogels (H). In some cases, an easy-dissolving gel is desirable; in other cases, a material resistant to dissolving and that guarantees integrity is preferred. The integrity of chitosan was significantly affected (*p* < 0.05) as the squid gelatin ratio increased (Table 1). The hydrogels without gelatin (HM1) declined by about 19.9%, which was remarkably less than in samples corresponding to the HM2 (26.7%) and HM3 (33.4%) hydrogels. The highest solubility of hydrogels with gelatin content can be attributed to the fact that gelatin is rich in the imino amino acid hydroxyproline [19], which produces a relaxation effect on the chitosan chain and increases its hydrophilic character.

### 2.3. Textural Profile

The textural parameters of the chitosan/gelatin hydrogels were determined by TPA (Table 2). The addition of squid gelatin resulted in a significant difference (*p* < 0.05) in the hardness of the chitosan hydrogel (H), with hardness decreasing from 1339.6 g force (HM1) to 1041.9 g force (HM3). Moreover, chewiness also decreased with increasing squid gelatin, from 1026.2 g force (HM1) to 917.2 g force (HM3).

On the other hand, the three obtained hydrogels were considered elastic because the values of springiness were close to one [20]. The addition of squid gelatin changed the elasticity of the chitosan hydrogels by improving the springiness significantly (*p* < 0.05) from 0.92% (HM1) to 0.97% (HM3). Additionally, it can be noted that the resilience of the chitosan hydrogel increased (*p* < 0.05) from 18.9% (HM1) to 35.6% (HM3). A previous study reported that the addition of chitosan to gelatin hydrogels reduced gel springiness and resilience [20]. This implies that the presence of squid gelatin in chitosan hydrogels leads to the hydrogel being more flexible and less rigid, making it appropriate for certain biomedical [21] or food [22] applications.

### 2.4. Appearance and Morphology

**Figure 2:**
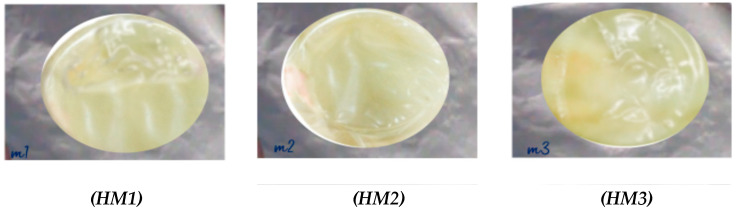
Photograph of the hydrogels: chitosan–squid gelatin mass ratio: 1:0 (HM1), 2:1 (HM2), and 1:2 (HM3). Water content: 25.1% ± 6.6 (HM1), 29.2% ± 6.3 (HM2), and 33.6% ± 6.9 (HM3).

**Figure 3:**
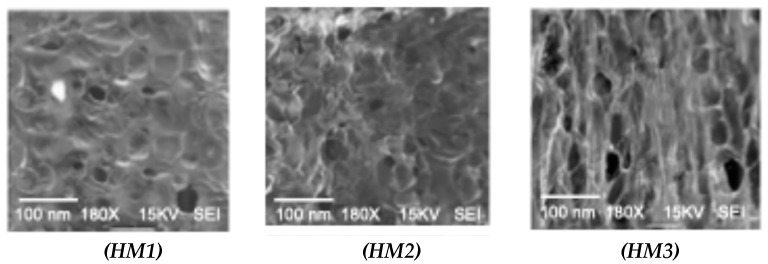
SEM images of the hydrogels’ morphology: chitosan–squid gelatin mass ratios: 1:0 (HM1), 2:1 (HM2), and 1:2 (HM3).

The macroscopic images of the hydrogels (Figure 2) indicate that the appearance of the HM1 hydrogels was more brittle and transparent than those containing gelatin (HM2 and HM3). Although the water content was not significantly different (*p* > 0.05), the M3 hydrogels were softer than those corresponding to HM2 and HM1. The SEM images (Figure 3) show big differences between the structures obtained for the different hydrogels. The M1 hydrogel had lower porosity, which corroborates the data obtained in the TAP results about hardness and chewiness values. Similar findings were reported in another study completed by Ge et al. [20]. In their study, the pore size in a gelatin hydrogel decreased after the incorporation of chitosan. The porosity and rougher surface could be associated with the increase in the crosslinking covalent and non-covalent bonding between hydrolysates and chitosan as the squid gelatin content increased; this would result in more significant intermolecular aggregation and, consequently, would produce some irregularities on the surface of the hydrogel. These results suggest that squid gelatin presents a valuable interaction with chitosan.

### 2.5. Antioxidant Activity

One mechanism involved in cell aging is the trapping of free radicals. Therefore, this work evaluated the antioxidant activity of hydrogels using two different methods, i.e., their capacity to trap radicals (DPPH assay) and to neutralize radical oxygen (ORAC assay).

The three hydrogels showed scavenging activity against DPPH and reactive oxygen species (ORAC assay) (Table 1). Hydrogels without gelatin showed some antioxidant activity, since it was reported that chitosan could trap radicals due to the presence of hydroxyl and amino groups [23]. The DPPH radical scavenging activity of the HM1, HM2, and HM3 conditions was measured to be 52.2%, 60.35%, and 71.49% at a concentration of 96 μg·mL^−1^. Additionally, the IC_50_ values of the hydrogels were ranked as follows: HM3 < HM2 < HM1. In the hydrogels containing gelatin, the antioxidant activity measured by the ORAC method increased up to three times more than in the one based on chitosan alone: HM3 (5.6 μmol TE·g^−1^) > HM2 (4.2 μmol TE·g^−1^) > HM1 (1.7 μmol TE·g^−1^). The Results obtained for HM2 and HM3 were higher than the IC_50_ DPPH value of commercial gelatin–chitosan hydrogel (8.30 μg·mL^−1^) [24], but the ORAC results were higher than those reported for silk sericin hydrogels (3.84 μmol TE·g^−1^) [25].

The increase in antioxidant activity in the chitosan hydrogels due gelatin addition could be explained on the basis of gelatin-inducing modifications in the chitosan properties [26]. During the transformation of collagen into gelatin, the collagen triple helix of the molecular structure unfolds. Collagen dissolves into random peptide chains, and its capacity to donate electrons could be due to the presence of amino acids such as glycine, proline, and hydroxyproline [27]; this presence, in conjunction with the positively charged amino groups of chitosan that could remain free, would have a synergistic effect against the free radicals and improve the antioxidant activity of the obtained hydrogels [28].

### 2.6. Chemical Characterization of Hydrogels

Chitosan polymerization with glutaraldehyde as a crosslinking agent in the presence of gelatin can form a network between both polymers [29]. Then, Fourier-transform infrared spectroscopy (FT-IR) and nuclear magnetic resonance (^1^H-NMR) analyses were performed to establish mainly the interactions between chitosan and squid gelatin.

#### 2.6.1. Fourier-Transform Infrared (FT-IR)

**Figure 4:**
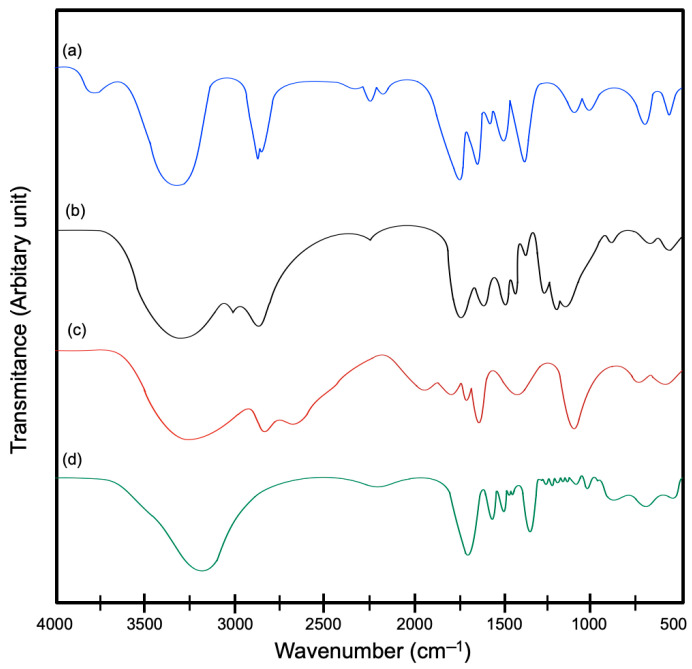
FT-IR spectra of (a) squid gelatin and (b) chitosan; squid gelatin mass ratios: (c) 1:0 (HM1) and (d) 1:2 (HM3). HM1 and HM3 contain glutaraldehyde (50%) at 1% (wt%).

The chitosan–squid gelatin hydrogel FT-IR spectrum (Figure 4) was comparable to that displayed by the squid gelatin, chitosan, and chitosan hydrogel (Table 3). Squid gelatin IR transmittance spectrum displayed the five major collagen characteristic peaks (Figure 4a) [30]. The peak associated with N-H stretching frequency (Amide A) was observed at around 3431 cm^−1^. The band observed at 2830 cm^−1^ is related to asymmetric stretch of CH_2_ and NH_3_^+^ (Amide B). The C=O stretching (Amide I) was detected at 1635 cm^−1^, whereas the N–H and C–N torsional vibration (Amide II) was observed at 1585 cm^−1^ and the absorption band around 1283 cm^−1^ (Amide III); this band is associated with CH residual groups. The wagging at 672–562 cm^−1^ is associated with N-H and C-OH out-of-plane bending [31]. Regarding chitosan, the spectra showed peaks at around 3260, 2835, 1645, and 1563 cm^−1^, corresponding to Amides A, B, I, and II, respectively [29]. Additionally, the skeletal vibrations typical of the chitosan structure appeared at 1080 cm^−1^, corresponding to the pyranosic and C-O-C groups. The band at 1480 cm^−1^ represents the vibrations of the -OH group of the primary alcohol group. In the region between 1406 and 1249 cm^−1^, peaks associated with -CH_2_ torsion and C-N tension vibration were observed. The last bands at 675 and 596 cm^−1^ are characteristic of glycosidic stretching [29]. The chitosan absorption bands associated with Amide I and Amide II may come from residues of the acetamide group after deacetylation [31].

The FT-IR spectra of the chitosan hydrogel showed that the peaks at 1320 and 1249 cm^−1^ disappeared, indicating that glutaraldehyde may hinder the two peaks. Moreover, the increase in the peak intensity at 1581 cm^−1^ can be attributed to the reaction of chitosan with glutaraldehyde [32]. Meanwhile, in the FT-IR spectra of the chitosan–squid gelatin–glutaraldehyde hydrogel, a slight shift of Amide A from 3260 to 3175 cm^−1^ was observed. This shift indicates an interaction between the N-H and O-H groups of the chitosan with the C=O group of the gelatin; this interaction may be due to Schiff base formation between the aldehyde group of glutaraldehyde and the free amino group of gelatin. Moreover, the -OH group peak decrease indicates that these groups were consumed during the crosslinking reactions with glutaraldehyde under acidic conditions [33]. In addition, the reduction in the amide II peak of chitosan and gelatin indicates the formation of hydrogen bond interaction among the groups belonging to chitosan (NH_2_ and OH) and squid gelatin (NH_2_, C=O, and OH) [29].

#### 2.6.2. Proton Nuclear Magnetic Resonance (^1^H-NMR)

**Figure 5:**
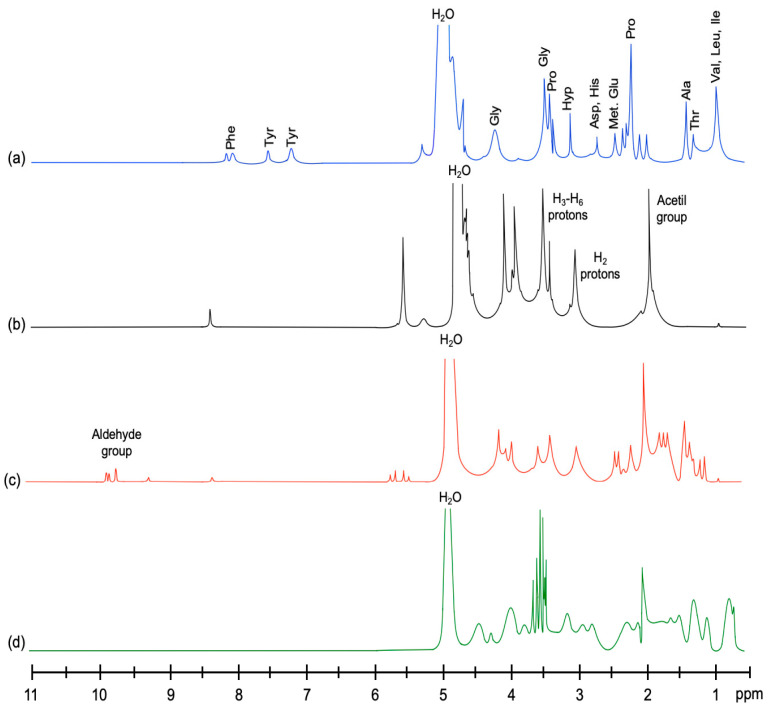
^1^H-NMR spectra of (a) squid gelatin and (b) chitosan; squid gelatin mass ratios: (c) 1:0 (HM1) and (d) 1:2 (HM3). HM1 and HM3 contain glutaraldehyde (50%) at 1% (wt%).

The ^1^H-NMR spectra of squid gelatin and chitosan were compared to previous studies. The ^1^H-NMR spectrum of the squid gelatin (Figure 5a) showed signals corresponding to the side chains of different amino acid protons [34]. The proton signals within the region of chemical shifts ranged from 0.5 to 1.5 ppm, which might be assigned to aliphatic carbon atoms of valine, leucine, and isoleucine. The signal centered at 4.2 ppm could be attributed to the glycine signal (CH_2_), while the signals at 2.2 and 3.7 ppm would correspond to proline and that at position 3.3 ppm would be attributed to hydroxyproline [35]. The prominent peak at 5.0 ppm indicates the presence of water molecules within the gelatin structure [36]. A weaker signal between 7.3 and 7.9 ppm is assigned to aromatic rings [37]. Regarding chitosan, this molecule showed a typical ^1^H-NMR spectrum (Figure 5b). The singlet peak at 1.9 ppm represents three protons of N-acetyl glucosamine (GlcNA), the peak at 3.1–3.2 ppm represents H_2_ protons of glucosamine (GlcN) residues, and the signals at 3.4–3.8 ppm represent protons of D-glucosamine (H_3_-H_6_ protons) [38].

The ^1^H-NMR spectrum of the chitosan hydrogel (Figure 5c) showed a reduction in the intensities of the peaks between 3.0 and 4.0 ppm and several new peaks that were not well defined at about 1.0–1.8 ppm. This behavior indicated that glutaraldehyde created an ionic environment, inducing the crosslinking between chitosan glucosamine groups and glutaraldehyde [39]. Meanwhile, the peak at about 9.6 ppm is associated with aldehyde groups [38].

In the spectrum of the chitosan–squid gelatin–glutaraldehyde hydrogel (Figure 5d), a slight shift in peaks associated with leucine, proline, methionine, and hydroxyproline protons was observed. In addition, the peaks associated with chitosan protons H_2_ and H_3_-H_6_ were not detected. In contrast, new peaks were observed between 3.4 and 4.5 ppm. This indicates that the GlcNA and GlcN protons of chitosan were modified due to the different bonds produced when gelatin complexed with chitosan and glutaraldehyde as a result of electrostatic interactions, hydrogen bond formation, and hydrophobic interactions. Such interactions could be explained on the basis of the presence of NH_2_, OH, and C=O groups included in each ingredient used to obtain the hydrogel. As discussed previously, the intermolecular interaction between chitosan and gelatin might determine the antioxidant activity of the resulting molecule.

## 3. Conclusions

Under the conditions of this study, it was established that the mixture resulting from squid by-product gelatin and chitosan rendered hydrogels with valuable structures due to the high compatibility of the two components. The addition of squid by-product gelatin significantly increased the springiness and resilience of the composite films. The hydrogel containing squid gelatin exhibited a higher antioxidant activity. In conclusion, squid by-product gelatin might be useful as a new source of additives in the preparation of functional hydrogels in composites including chitosan, leading to remarkable antioxidant properties.

The composite of commercial chitosan and squid by-product gelatin presents the possibility of producing a new material with potential applications in the biomedical or food industries. Nevertheless, the resulting properties and, consequently, possible applications of the hydrogels will depend on the concentration of each component. Therefore, different mixture proportions, as well as the search for further mechanical and antimicrobial properties, among others, are important and could be the basis for future research.

## 4. Materials and Methods

### 4.1. Materials

Squid (Dosidicus gigas) by-products (head with arms and tentacles) were obtained from a local squid processing plant in Guaymas, Sonora, Mexico, and used as a source of collagen. They were transported immediately to the laboratory and skin was removed. Afterward, they were mixed, cut, divided into 100 g portions, placed in high-density polyethylene bags, and frozen at −20 °C until use. The chitosan used in the work was of commercial origin, extracted from crab shells, with 85% deacetylation and high molecular weight, and purchased from Sigma (Chemical Co., Toluca, Mexico). Glutaraldehyde (50%) and all other reagents were of analytical grade, from Sigma.

### 4.2. Gelatin Extraction

Gelatin extraction followed the methodology described previously [14], with some modifications. Squid tissues were chopped and washed with distilled water and placed in a NaOH solution (0.5 M), ratio 1:3 (*w/v*), for 90 min at 25 °C. The alkali-treated tissues were washed with plenty of water until the washing liquid had a pH close to neutrality (pH < 7.5). Subsequently, the tissues were introduced to an HCl solution (0.2 M), ratio 1:3 (*w/v*), for 180 min at 25 °C. In order to transform the collagen into gelatin, the tissues were immersed in hot water (65 °C) for 12 h with constant stirring to a 1:4 (*w/v*) ratio. Then, the mixture was filtered using a double layer of gauze cloth. The resulting product was allowed to stand at 4 °C for 2 days until gelation occurred. Freeze-dried gelatin was used as a raw material to produce hydrogels. The presence of collagen in the gelatin obtained was confirmed by detecting hydroxyproline (9.3 g·100 g^−1^).

### 4.3. Chitosan–Gelatin Hydrogel

The hydrogels were prepared as described in previous research, with some modifications [3,40]. Chitosan (1%, wt/vol) and squid gelatin (1% wt/vol) solutions were separately prepared by dissolving the chitosan or lyophilised squid gelatin in 0.1 M acetic acid at 25 °C, with mechanical stirring overnight. The two solutions were stirred for 60 min, stirred again for 30 min, and degassified under vacuum. The final chitosan–squid gelatin mass ratios were 1:0 (M1), 2:1(M2), and 1:2 (M3). The crosslinking reagent, glutaraldehyde (50%), was slowly added to the gel solution precursors under constant stirring. The final concentration of glutaraldehyde in the solutions was 1% (wt%). Furthermore, 30–40 mL of the three hydrogels were poured into Petri dishes and dried at 25 °C under vacuum conditions for 72 h. After polymerization, the prepared hydrogels were soaked in a large amount of water for solvent exchange [41]. The water was exchanged every 24 h, and the equilibrated hydrogels (HM1, HM2, and HM3) were obtained after 72 h.

### 4.4. Analysis

#### 4.4.1. Viscosity Determination of Hydrogel-Forming Solutions

A prior step to forming the hydrogels is preparing hydrogel-forming solutions [42]. For this, the M1-M3 were solutions subjected to a shear test in a stable state using a modular compact rheometer (MCR; model 102) equipment (Anton Paar GmbH, Graz, Austria), utilizing concentric cylinder geometry. The shear rate employed ranged from 100/s to 500/s at 25 °C. The viscosity value was reported as the average value of 30 measurement points for 500 s and expressed in centipoise second (cPa·s).

#### 4.4.2. Water Content and Hydrogel Stability in Aqueous Solution

The water content (q) and stability of the hydrogels (W) in an aqueous medium was determined by weighing 10 mg samples and drying at 100 °C for 24 h. The dried samples were placed in 50 mL of a 1M TRIS buffer solution at pH 7.2, which contained 0.02% *w/v* sodium azide (to prevent microbial growth), and then shaken for 24 h. Subsequently, they were removed from the medium and dried at 100 °C for 24 h to determine the weight of the dry matter that was not dissolved in the medium [43]. Each sample was analyzed in quintuplicate. The water content and degree of solubility were calculated according to Equation (1) and Equation (2), respectively.

(1) $$q=\left[\frac{\mathrm{Wf}-\mathrm{Wi}}{\mathrm{Wi}}\right]\times 100$$

(2) $$W=\left[\frac{\mathrm{Wf}-\mathrm{Wm}}{\mathrm{Wf}}\right]\times 100$$

where Wi is the initial weight (g) of sample, Wf is the weight of the dry matter (g), and Wm is the weight of the dry matter that did not dissolve after 24 h.

#### 4.4.3. Textural Profile

Texture profile analysis of hydrogels was carried out by using a Texture Analyser (TA-XT Plus Stable Micro Systems Ltd., Hamilton, MA, USA) equipped with a 100-N load cell. The test was performed with a returned speed of 7 mm·s^−1^ and a force of 100 N. A double compression cycle test was performed at 40% deformation using an aluminum cylinder probe (SMS P/25, 25 mm diameter). The time elapsed between the two compression cycles was 3 s. The textural profile analysis was estimated by measuring the maximum effort required to cut the gel (hardness), springiness (elasticity), and deformation recovery (resilience) by compressing each sample between stainless steel plates. The TPA values were calculated from the resulting force–deformation plots.

#### 4.4.4. Surface Morphology of Hydrogels

Scanning electron microscopy (SEM) was used to observe the surface morphology of the chitosan and chitosan-squid gelatin hydrogels. Dry hydrogels were coated with a thin layer of carbon paper (13 mm) and 20 mm gold coating before being imaged by using SEM equipment (JEOL 5410LV, Peabody, MA, USA) at 15 kV of acceleration voltage.

#### 4.4.5. Hydrogel Antioxidant Activity

The hydrogel antioxidant activity was evaluated by the DPPH radical scavenging test [44] and the reactive oxygen species assay (ORAC) [45].

The DPPH assay quantifies the reduction of the 2,2-diphenyl-1-picrylhydrazyl radical in 2,2-diphenyl-1-pricryl hydrazine due to the antioxidant action of compounds that contain hydroxyl groups, which discolor the reagent. The mechanism evaluated by the DPPH assay is hydrogen transfer (HAT). For this test, 20 µL of the sample was used, and 200 µL of DPPH solution (1.25 mg/50 mL methanol) was added. The absorbance (Abs) was determined at 30 min at a wavelength of 515 nm using an UV spectrophotometer (Thermo Scientific, Multiskan, GO, USA). The percentage of inhibition of the DPPH radical was calculated according to Equation (3), in which Ac and Ah represent the Abs of the control (DPPH solution) and the hydrogels, respectively.

(3) $$\mathrm{Scavening}\;\left(\%\right)=\left[\frac{\mathrm{Ac}-\mathrm{Ah}}{\mathrm{Ac}}\right]\times 100$$

The concentration of the sample (μg·mL^−1^) needed to inhibit 50% of the DPPH radical (IC_50_) was also determined by employing an inhibition curve established from absorbance values obtained from different concentrations of hydrogels.

The ORAC method was performed by evaluating the loss of fluorescein fluorescence for 90 min at 37 °C in the presence of 2,2′-azobis (2-amidinopropane) dihydrochloride (AAPH). Each sample (0.5 mg·mL^−1^) was compared to a standard curve to express the results as equivalents of Trolox (6-hydroxy-2,5,7,8-tetramethylchroman-2-carboxylic acid). The results were expressed as μmol TE·g^−1^ sample.

#### 4.4.6. Chemical Characterization of Hydrogels

The interactions between chitosan and gelatin were established using infrared spectroscopy and nuclear magnetic resonance. The spectrophotometric studies were carried out on the following materials in a dry state: chitosan, gelatin, and the hydrogels from treatments M1 and M3.

- Fourier-Transform Infrared Spectroscopy (FT-IR)

The spectrum of the lyophilized hydrogels (1 mg in 100 mg potassium bromide) was obtained at 24 ± 1 °C on a Perkin Elmer spectrometer (Frontier MIR/FIR, Walthman, MA, USA). The spectra were collected between 4000 and 400 cm^−1^ at a resolution of 4 cm^−1^, accumulating 16 scans per spectrum. During spectral acquisition, the system was purged with nitrogen.

- Nuclear magnetic resonance (^1^H-NMR)

^1^H-NMR spectra were acquired at 24 ± 1 °C using a Bruker Avance 400 nuclear magnetic resonance spectrometer (Billerica, MA, USA) operating at 400 MHz. Lyophilized samples (1 mg) were dissolved in 0.5 mL of deuterated water (D_2_O) and 1% (*v*/*v*) deuterated hydrochloride acid solution (DCl 40% in D_2_O). Dimethylsilapentane sulfonic acid was used as a reference. The spectra window was 20 ppm.

### 4.5. Statistical Analysis

A completely randomized design was applied. For the analysis of viscosity, stability, and antioxidant activity in an aqueous medium, a one-way analysis of variance (ANOVA) was applied. Tukey’s mean comparison test established differences between means at a 95% significance level (α = 0.05). The PASW Statistics 18 software for Windows (SPPS Inc., Chicago, IL, USA).

## Figures and Tables

**Table 1:** Hydrogel-forming solutions viscosity (μ), hydrogel stability in aqueous solution at pH 7.2 (W), and hydrogel antioxidant activity (DPPH and ORAC assays).

Assay	M1	M2	M3
μ (cPa·s) ^1^	308.9 ^a^ ± 1.7	103.9 ^b^ ± 1.4	78.6 ^c^ ± 1.2
W (%) ^2^	19.9 ^c^ ± 1.4	26.7 ^b^ ± 2.4	33.4 ^a^ ± 1.9
DPPH (IC_50_; μg/mL)	30.7 ^c^	26.1 ^b^	22.4 ^a^
ORAC (μmol TE/g) ^3^	1.7 ^c^ ± 0.3	4.2 ^b^ ± 0.2	5.6 ^a^ ± 0.5

Chitosan–squid gelatin mass ratios: 1:0 (M1), 2:1 (M2), and 1:2 (M3). All mixtures contain glutaraldehyde (50%) at 1% (wt%). ^1^ Mean value ± standard deviation from 30 measurement points. ^2^ Mean value ± standard deviation from five separate samples. ^3^ Mean value ± standard deviation from three separate samples. In each row, means values followed by different letters (a, b, c) indicate significant differences (*p* < 0.05).

**Table 2:** Textural properties of the chitosan–squid gelatin hydrogel.

Textural Properties ^1^	HM1	HM2	HM3
Hardness (g force)	1339.6 ^a^ ± 29.7	1170.3 ^b^ ± 20.1	1041.9 ^c^ ± 18.8
Springiness (%)	0.92 ^c^ ± 0.04	0.95 ^b^ ± 0.06	0.97 ^a^ ± 0.01
Chewiness (g force)	1026.2 ^a^ ± 3.2	1007.4 ^b^ ± 6.1	917.2 ^c^ ± 8.6
Resilience (%)	18.9 ^b^ ± 1.8	34.2 ^a^ ± 3.1	35.6 ^a^ ± 2.1

Chitosan–squid gelatin mass ratios: 1:0 (HM1), 2:1 (HM2), and 1:2 (HM3). All hydrogels contain glutaraldehyde (50%) at 1% (wt%). ^1^ Mean value ± standard deviation from five separate samples. In each row, mean values followed by different letters (a, b, c) indicate significant differences (*p* < 0.05).

**Table 3:** Assignment of FTIR spectra of absorption bands of the squid by-product gelatin, chitosan, chitosan (HM1) hydrogel, and chitosan–squid gelatin hydrogel (HM3).

Assignments	Squid Gelatin	Chitosan	Chitosan Hydrogel	Chitosan–Squid Gelatin Hydrogel
N-H stretching, Amide A	3431	3260	3250	3175
CH_2_ and NH_3_^+^asymmetric stretch, Amide B	2830	2835	2750	–
C=O stretching, Amide I	1635	1645	1635	1630
N–H and C–N torsional vibration, Amide II	1585	1581	1581	1545
CH residual groups, Amide III	1283	–	–	1285
Primary alcohol OH group	1480	1480	1406	1480
-CH_2_ torsion and C-N tension vibration	–	1406–1249	–	–
Pyranosic and C-O-C groups	–	1080	1050	–
N-H and C-OH out-of-plane bending	672–562	675–564	670–549	665–550

## Data Availability

The data presented in this study are available in the article. Further information is available upon request from the corresponding authors.

## References

[1] Ho T.-C., Chang C.-C., Chan H.-P., Chung T.-W., Shu C.-W., Chuang K.-P., Duh T.-H., Yang M.-H., Tyan Y.-C. (2022). Hydrogels: Properties and applications in biomedicine. Molecules.

[2] Jagan D., Kalim D., Deon B., Yi-Cheun Y., Jagan D., Kalim D., Deon B. (2023). Hydrogels: Definition, History, Classifications, Formation, Constitutive Characteristics, and Applications. Multicomponent Hydrogels: Smart Materials for Biomedical Applications.

[3] Peng Z., Peng Z., Shen Y. (2011). Fabrication and properties of gelatin/chitosan composite hydrogel. Polim. Plast. Technol. Eng..

[4] El Bourakadi K., Qaiss A., Bouhfid R., Sabu T., Bhasha S., Purnima J., Shashank S. (2023). Sustainable hydrogels in food packaging systems. Sustainable Hydrogels Synthesis, Properties, and Applications.

[5] Younes I., Rinaudos M. (2015). Chitin and chitosan preparation from marine sources. Structure, properties and applications. Mar. Drugs.

[6] Srinivasa P.C., Tharanathan R.N. (2007). Chitin/chitosan-safe, ecofriendly packaging materials with multiple potential uses. Food Rev. Int..

[7] Mathew S.A., Arumainathan S. (2022). Crosslinked Chitosan−gelatin biocompatible nanocomposite as a neuro drug carrier. ACS Omega.

[8] Alipal J., Mohd Pu’adA N.A.S., Lee T.C., Nayan N.H.M., Sahari N., Basri H., Idris M.I., Abdullah H.Z. (2021). A review of gelatin: Properties, sources, process, applications, and commercialization. Mater. Today.

[9] Uranga J., Etxabide A., Cabezudo S., de la Caba K., Guerrero P. (2020). Valorization of marine-derived biowaste to develop chitin/fish gelatin products as bioactive carriers and moisture scavengers. Sci. Total Environ..

[10] Squid Market Analysis APAC, Europe, North America, South America, Middle East and Africa—China, India, Japan, Spain, Peru—Size and Forecast 2024–2028. https://www.technavio.com/report/squid-market-industry-analysis.

[11] FAO Food and Agriculture Organization of the United Nations. The State of World Fisheries and Aquaculture 2024..

[12] Squid and Squid By-products. Crops. https://www.ams.usda.gov/sites/default/files/media/Squid-TR-011216.pdf.

[13] Uriarte-Montoya M.H., Santacruz-Ortega H., Cinco-Moroyoqui F.J., Rouzaud-Sández O., Plascencia-Jatomea M., Ezquerra-Brauer J.M. (2011). Giant squid skin gelatin: Chemical composition and biophysical characterization. Food Res. Int..

[14] Chan-Higuera J.E., Robles-Sánchez R.M., Burgos-Hernández A., Márquez-Ríos E., Velázquez-Contreras C.A., Ezquerra-Brauer J.M. (2016). Squid by-product gelatines: Effect on oxidative stress biomarkers in healthy rats. Czech J. Food Sci..

[15] Ezquerra-Brauer J.M., Aubourg S.P. (2019). Bioactive peptides from collagen hydrolysates from squid (Dosidicus gigas) by-products fractionated by ultrafiltration. Int. J. Food Sci. Technol..

[16] Salomé Machado A.A., Martins V.C.A., Plepis A.M.G. (2002). Thermal and rheological behavior of collagen. Chitosan Blends. J. Therm. Anal. Cal..

[17] Do Amaral Sobral P.J., Gebremariam G., Drudi F., De Aguiar Saldanha Pinheiro A.C., Romani S., Rocculi P., Dalla Rosa M. (2022). Rheological and viscoelastic properties of chitosan solutions prepared with different chitosan or acetic acid concentrations. Foods.

[18] Bertolo M.R.V., Martins V.C.A., Plepis A.M.G., Bogusz S. (2021). Rheological study of the incorporation of grape seed extract in chitosan and gelatin coatings. J. Appl. Polym. Sci..

[19] Giménez B., Alemán A., Montero P., Gómez-Guillén M.C. (2009). Antioxidant and functional properties of gelatin hydrolysates obtained from skin of sole and squid. Food Chem..

[20] Ge S., Liu Q., Li M., Liu J., Lu H., Li F., Zhang S., Sun Q., Xiong L. (2018). Enhanced mechanical properties and gelling ability of gelatin hydrogels reinforced with chitin whiskers. Food Hydrocoll..

[21] Goder Orbach D., Sharabani-Yosef O., Hadad O., Zilberman M. (2024). Gelatin-based polymers can be processed to highly resilient biocompatible porous hydrogel scaffolds for soft tissue regeneration applications. Gels.

[22] Khorshidi S., Khoobbakht F., Mirmoghtadaie L., Hosseini S.M. (2023). Characterization of gellan gum-chitosan based hydrogel to evaluate as a potential gelatin substitute. Food Hydrocoll..

[23] Muthu M., Gopal J., Chun S., Devadoss A.J.P., Hasan N., Sivanesan I. (2021). Crustacean Waste-Derived Chitosan: Antioxidant Properties and Future Perspective. Antioxidants.

[24] Mirjalili F., Mahmoodi M. (2023). Controlled release of protein from gelatin/chitosan hydrogel containing platelet-rich fibrin encapsulated in chitosan nanoparticles for accelerated wound healing in an animal model. Int. J. Biol. Macromol..

[25] Baptista-Silva S., Bernardes B.G., Borges S., Rodrigues I., Fernandes R., Gomes-Guerreiro S., Pinto M.T., Pintado M., Soares R., Costa R. (2022). Exploring silk sericin for diabetic wounds: An in situ- forming hydrogel to protect against oxidative stress and improve tissue healing and regeneration. Biomolecules.

[26] Sinkowska A., Wisniewski M., Skopinska J., Kennedy C.J., Wess T.J. (2004). The photochemical stability of collagen–chitosan blends. J. Photochem. Photobiol. A Chem..

[27] Mendis E., Rajapakse N., Kim S.K. (2005). Antioxidant properties of a radical scavenging peptide purified from enzymatically prepared fish skin gelatin hydrolysates. J. Agri. Food Chem..

[28] Ngo D.H., Kim S.K. (2014). Antioxidant effects of chitin, chitosan, and their derivatives. Adv. Food Nutr. Res..

[29] Staroszczyk H., Sztuka K., Wolska J., Wojtasz-Pająk A., Kolodziejska I. (2014). Interaction of fish gelatin and chitosan in uncrosslinked and crosslinked with EDC films: FT-IR study. Spectrochim. Acta A Mol. Biomol. Spectrosc..

[30] Riaz T., Zeeshan R., Zarif F., Ilyas K., Muhammad N., Safi S.Z., Rahim A., Rizvi S.A.A., Rehman I.U. (2018). FTIR analysis of natural and synthetic collagen. Appl. Spectrosc. Rev..

[31] Brugnerotto J., Lizardi J., Goycoolea F., Argüelles W., Desbrières J. (2001). An infrared investigation in relation with chitin and chitosan characterization. Polymer.

[32] Li B., Shan C.-L., Zhou Q., Fang Y., Wang Y.-L., Xu F., Han L.-R., Ibrahim M., Guo L.-B., Xie G.-L. (2013). Synthesis, Characterization, and Antibacterial Activity of Cross-Linked Chitosan-Glutaraldehyde. Mar. Drugs.

[33] Patel S., Srivastava S., Singh M.R., Singh D. (2018). Preparation and optimization of chitosan-gelatin films for sustained delivery of lupeol for wound healing. Int. J. Biol. Macromol..

[34] Nguyen D., Chen C., Pettitt B.M., Iwahara J. (2019). NMR methods for characterizing the basic side chains of proteins: Electrostatic interactions, hydrogen bonds, and conformational dynamics. Methods Enzymol..

[35] Rodin V.V., Izmailova V.N. (1996). NMR method in the study of the interfacial adsorption layer of gelatin. Coll. Surf. A Physicochem. Eng. Aspcects.

[36] Fullerton G.D., Nes E., Amurao M., Rahal A., Krasnosselskaia L., Cameron I. (2006). An NMR method to characterize multiple water compartments on mammalian collagen. Cell Biol. Int..

[37] Sell D.R., Monnier V.M. (1989). Structure elucidation of a senescence cross-link from human extracellular matrix: Implication of pentoses in the aging process. J. Biol. Chem..

[38] López-Cervantes J., Sánchez-Machado D.I., Sánchez-Duarte R.G., Correa-Murrieta M.A. (2018). Study of a fixed-bed column in the adsorption of an azo dye from an aqueous medium using a chitosan–glutaraldehyde biosorbent. Adsorpt. Sci. Technol..

[39] Monteiro O.A., Airoldi C. (1999). Some studies of crosslinking chitosan-glutaraldehyde interaction in a homogeneous system. Int. J. Biol. Macromol..

[40] Franco R.A., Nguyen T.H., Lee B.Y. (2011). Preparation and characterization of electrospun PCL/PLGA membranes and chitosan/gelatin hydrogels for skin bioengineering applications. J. Mat. Sci. Mater. Med..

[41] Chen F., Zhou D., Wang J., Li T., Zhou X., Gan T., Handschuh-Wang S., Zhou X. (2018). Rational fabrication of anti-freezing, non-drying tough organohydrogels by one-pot solvent displacement. Angew. Chem. Int. Ed..

[42] Zamudio-Flores P.B., García-Amezquita L.E., Ramos-Martínez A., Tirado Gallegos J.M., Bello-Pérez L.A., Salgado-Delgado R. (2013). Soluciones formadoras de películas a base de almidón oxidado de avena mezclada con quitosano: Caracterización reológica y propiedades mecánicas de sus películas. Rev. Iberoam. Pol..

[43] Gontard N., Guilbert S., Cuq J.L. (1992). Edible wheat gluten films influence of the main process variables on film properties using response surface methodology. J. Food Sci..

[44] Brand-Williams W., Cuvelier M.E., Berset C. (1995). Use of a radical method to evaluate antioxidant activity. LWT-Food Sci. Technol..

[45] Prior R.L., Hoang H., Gu L., Wu X., Bacchiocca M., Howard L., Hampsch-Woodill M., Huang D., Ou B., Jacob R. (2003). Assays for hydrophilic and lipophilic antioxidant capacity (oxygen radical absorbance capacity (ORACFL) of plasma and other biological and food samples. J. Agr. Food Chem..

